# Influence of Fiber Orientation on Mechanical Response of Jute Fiber-Reinforced Polymer Composites

**DOI:** 10.3390/polym16182573

**Published:** 2024-09-11

**Authors:** Roberto Iquilio, José Luis Valín, Kimio Villalobos, Sergio Núñez, Álvaro González, Meylí Valin

**Affiliations:** 1 Escuela de Ingeniería Mecánica, Pontificia Universidad Católica de Valparaíso, Av. Los Carrera 01567, Quilpué 2430000, Chile; jose.valin@pucv.cl (J.L.V.); kimio.villalobos.m@mail.pucv.cl (K.V.); sergio.nunez@pucv.cl (S.N.); alvaro.gonzalez.o@pucv.cl (Á.G.); 2 Departamento de Ingeniería Mecánica, Universidad de Concepción, Concepción 4030000, Chile; mvalin@udec.cl

**Keywords:** polymer composite, fiber orientation, DIC

## Abstract

The influence of fiber orientation on the mechanical behavior of a polymer matrix composite reinforced with natural jute fibers is investigated in this study. Two fiber orientation configurations are examined: the first involves woven fibers aligned in the direction of testing, while the second considers a 45° orientation. The research involves manufacturing composite plates using jute fabric with the mentioned orientations, followed by cutting rectangular specimens for tensile testing to determine which orientation yields superior properties. Displacement fields are measured using a digital image correlation technique, synchronized with load data obtained from a universal testing machine equipped with a load cell to obtain stress–strain curves for each configuration. Results indicate that 0° specimens achieve higher stress but lower strain compared to 45° specimens. This research contributes to understanding the optimal fiber alignment for enhancing the mechanical performance of fiber-reinforced polymer composites.

## 1. Introduction

In recent years, increasing concerns about environmental sustainability and the impacts of global warming have driven the search for more eco-friendly materials in industrial applications. Among these materials, natural fibers have garnered significant attention due to their renewable nature, biodegradability, and lower environmental footprint compared to synthetic fibers. Natural fibers such as jute, flax, and hemp have been identified as promising reinforcements for polymeric matrices, providing a balance between mechanical performance and environmental benefits.

Jute, in particular, is a bast fiber derived from plants of the Tiliaceae family, with Corchorus olitorius and Corchorus capsularis being the most commonly cultivated species. These fibers are primarily composed of cellulose (61–73%), hemicellulose (13.6–23%), and lignin (12–16%), with small amounts of other components like waxes, pectin, and lipids. The chemical composition of jute fibers can vary depending on factors such as the soil conditions, the season of harvest, and the retting methods used (water, dew, or enzymatic). This variability in chemical composition, along with the natural variability inherent in plant fibers, poses challenges in consistently predicting the mechanical properties of jute-reinforced composites.

Several studies have examined the mechanical properties of polymer composites reinforced with jute fibers, highlighting both their potential and their limitations. Pervaiz and Sain [1], using non-woven mats of hemp fiber and polypropylene matrix, studied the performance of hemp-based natural fiber mat thermoplastic (NMT) by quantifying carbon storage potential and CO_2_ emissions and comparing the results with commercially available glass fiber composites. Elbadry et al. [2] conducted an extensive study on hybrid polymer matrix composites reinforced with jute fibers, investigating both fabric and mat forms. Their results showed that jute fibers significantly enhanced the tensile and flexural properties of the polymer matrix, although challenges such as incompatibility with hydrophobic polymer matrices and susceptibility to moisture absorption were noted. Similarly, Deb et al. [3] found that jute-polyester composites exhibited promising tensile and impact strength, suggesting that with proper treatment, these composites could serve as viable substitutes for glass fiber composites in applications where environmental benefits are prioritized. Munikenche Gowda et al. [4] demonstrated that jute fabric reinforcement significantly enhances the mechanical properties of polyester composites, improving the mechanical properties with chemical treatment of jute fiber [5,6] and the effects of fiber length [7].

Further studies, such as those by Shah et al. [8], explored the mechanical properties of jute-reinforced plastics (JRP) and hybrid composites of jute and glass fibers. Their findings indicated that while jute-reinforced laminates improved mechanical properties compared to the polymer matrix alone, they still fell short when compared to glass fiber-reinforced plastics. However, hybrid composites of jute and glass fibers showed a more balanced improvement in strength and modulus, highlighting the potential of jute as a cost-effective filler fiber in applications where the primary requirements for strength and modulus are moderate.

The review work by Summerscales et al. [9,10,11] offered a comprehensive overview of bast fibers, including jute, focusing on their mechanical properties, manufacturing techniques, and potential applications. Their analysis pointed out that bast fibers, including jute, have the potential to achieve a Young’s modulus comparable to that of synthetic fibers. However, predicting the mechanical properties of natural fiber composites remains challenging due to the variability in the fibers’ properties. They also explored the use of statistical modeling approaches, such as the Weibull distribution, to better predict the strength and failure modes of these composites, which is critical for their application in demanding sectors like automotive and construction.

Research by Faruk et al. [12] and Bledzki and Gassan [13] has further explored the development of biocomposites reinforced with natural fibers, emphasizing the importance of fiber treatment methods in enhancing fiber-matrix adhesion. Treatments such as silane coupling agents, alkaline treatment, and acetylation have been shown to improve the interfacial bonding between jute fibers and polymer matrices, leading to improved mechanical properties. Despite these advances, challenges such as the fibers’ moisture sensitivity remain a significant hurdle to their broader adoption in high-performance applications.

One of the critical factors influencing the mechanical performance of jute fiber-reinforced composites is the orientation of the fibers within the polymer matrix. As highlighted by Ku et al. [14], the tensile properties of these composites are strongly dependent on the interfacial adhesion between the fibers and the matrix, and the orientation of the fibers plays a crucial role in this interaction. Fiber orientation not only affects tensile strength but also impacts the distribution of deformations under load, which is vital for maintaining the structural integrity of the composite material. Ahmed and Vijayarangan [15] present an experimental investigation into the mechanical performance of woven jute-fabric-reinforced isothalic polyester composites. The study concludes that while the woven jute-fabric-reinforced polyester composites exhibit good mechanical properties and potential for medium load-bearing applications, their susceptibility to moisture absorption remains a challenge. Studies by Roe and Ansell [16] have shown that uniaxially oriented jute fibers can significantly enhance tensile strength and Young’s modulus, particularly at higher fiber volume fractions. However, the uneven distribution of deformations in composites with fibers oriented at 0 degrees often leads to premature fracture of the polymer matrix, especially in applications requiring high structural strength and durability.

Recent research has highlighted the use of natural fiber-reinforced composites and their impact on various mechanical properties. Arya et al. [17] indicated that the incorporation of PET100 foams as core materials in jute sandwich composites led to a substantial improvement in their flexural properties. Habib et al. [18] reported that hybrid composites incorporating densely packed short fibers and plain weave structures demonstrated the best mechanical properties, particularly in flexural strength and impact energy absorption. Islam et al. [19] concluded that alkali treatment facilitated a more compact composite structure, which accelerated crack propagation under impact loads, resulting in reduced impact resistance. Kumpati et al. [20] showed that natural jute fiber performed well when combined with glass fiber in a hybrid material. Lastly, Majumder et al. [21,22] demonstrated that although the addition of jute fibers led to improvements in ductility and energy absorption capacity, it resulted in a decrease in the flexural and compressive strength of the composite mortars, but the jute fiber nets with a denser mesh configuration showed a significant increase in stiffness and a greater capacity for energy absorption.

The issue of deformation concentration is particularly critical in structural applications, where localized stress can significantly reduce the lifespan of the material. The hypothesis proposed in this study is that a diagonal fiber orientation allows for a more even distribution of deformations, preventing their concentration and thereby reducing the likelihood of matrix fracture. This approach is essential for enhancing the strength and durability of composite materials in various industrial applications.

This article aims to conduct a comprehensive analysis of the influence of fiber orientation on the mechanical response of jute fiber-reinforced polymer composites. Comparative experimental tests were performed on samples with fiber orientations at 0 degrees and 45 degrees, documenting the mechanical behavior of each under various loading conditions. The results were analyzed to evaluate how fiber orientation affects the concentration of deformations and, consequently, the structural integrity of the material. The findings of this study confirm that diagonal fiber orientation enables a more uniform distribution of deformations, contributing to improved strength and preventing premature failure of the polymer matrix. These results validate the proposed hypothesis and offer valuable insights for the design of more durable and reliable composite materials in environmentally conscious engineering applications.

## 2. Materials and Methods

### 2.1. Materials

In this work, the effect of jute fiber orientation reinforcing a vinyl ester polymer matrix on the mechanical response of the material is studied.

**Figure 1:**
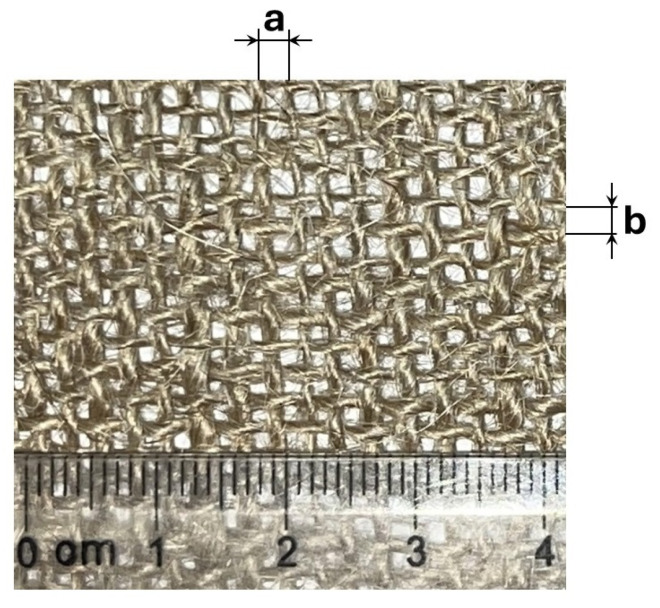
Schematic diagram of the warp and weft orientation in jute fabric.

Figure 1 shows the jute fabric used in the samples. It is important to note that neither the jute fiber nor the fabric is homogeneous. For this reason, a total of 15 measurements were taken for the distances between fibers *a* and *b*, as well as the fiber diameter *D*, with the respective standard deviations shown in parentheses. The results are as follows: a= 2.341 mm (0.211), b=2.297 mm (0.354) and D=1.154 mm (0.282).

Table 1 provides a summary of the quantities (by mass, expressed in grams) of jute fiber, resin, accelerator, and catalyst used to fabricate each plate from which the specimens to be tested in this study are cut. Both orientations (i.e., those aligned at 0 and 45 degrees) consist of four stacked layers of jute fiber, resulting in an average jute reinforcement percentage of 17%.

To fabricate composite material plates, the following steps were followed: 165 g of resin were added to a beaker, poured slowly to avoid air bubbles. Then, 0.66 g of accelerator were added and mixed slowly and thoroughly to ensure homogeneity. Next, 4.95 g of catalyst were added and mixed until a homogeneous mixture was achieved. Once the resin, accelerator, and catalyst mixture were ready, half of it was added to the mold and spread over all surfaces. The first layer of fiber was then added and pressed into the mold to ensure it was fully impregnated with resin. This step was repeated with additional layers of fiber and resin until all four required layers were added. The mold was then closed and placed in a hydraulic press under a pressure of approximately 1 ton for 24 h. After this time, the molds were removed from the press and opened to extract the composite material plates. The curing process was carried out by placing the plates in a forced convection oven at 50 °C for 24 h.

Using the methodology described earlier, two types of plates were fabricated: the first type consists of four layers of jute fabric reinforcement stacked with all layers oriented in the same direction, at 0 degrees (hereafter referred to as “Type A” specimens). The second type consists of plates with four layers of jute fabric reinforcement stacked with all layers oriented at 45 degrees (hereafter referred to as “Type B” specimens). Specimens for subsequent testing will be cut from these plates.

### 2.2. Methodology

The mechanical characterization was conducted in accordance with the ASTM D3039 [23], specifically focusing on tensile properties. Samples were prepared with a width of 12.5 mm, following the sheet-type specifications outlined in the standard. A universal tensile testing machine, Model WDW-200E (Jinan Kason Testing Equipment Co., Ltd., Jinan, Shandong, China), with a capacity of 200 kN, was used for the tests. The tests were conducted under displacement control at a grip speed of 2.0 mm/min, and the force was recorded using a load cell.

The displacement field within the specimen is obtained using the digital image correlation technique. For this, the specimens are pre-painted: a first layer of white paint is applied to the exposed surface of the specimen, followed by the application of a random pattern of fine black paint speckles. The test is recorded with a full HD camera, and the images are then extracted at one-second intervals. The image correlation analysis is performed using the NCORR software v1.2 [24].

NCORR calculates the deformations from the displacement gradients. This is accomplished using the Lagrangian approach, which is applied to finite solids as shown in Equations (1)–(3).

(1) $$E_{xx}=\frac{\partial u}{\partial x}+\frac{1}{2}\left({\left(\frac{\partial u}{\partial x}\right)}^{2}+{\left(\frac{\partial v}{\partial x}\right)}^{2}\right)$$

(2) $$E_{xy}=\frac{1}{2}\left(\frac{\partial u}{\partial y}+\frac{\partial v}{\partial x}+\frac{\partial u}{\partial x}\frac{\partial u}{\partial y}+\frac{\partial v}{\partial x}\frac{\partial v}{\partial y}\right)$$

(3) $$E_{yy}=\frac{\partial v}{\partial y}+\frac{1}{2}\left({\left(\frac{\partial u}{\partial y}\right)}^{2}+{\left(\frac{\partial v}{\partial y}\right)}^{2}\right)$$

The component $E_{xx}$, as stated in Equation (1), corresponds to the longitudinal strain, while the component $E_{xy}$, described in Equation (2), represents the angular strain. Additionally, the component $E_{yy}$, detailed in Equation (3), refers to the transverse strain.

At the end of the test, two independent signals are obtained:

- Force over time is recorded from the load cell of the universal testing machine.
- Strain tensor over time is recorded on the surface of the specimen through digital image correlation analysis.

Since both signals are temporally independent (as the acquisition of each signal is conducted separately) but correspond to the same test, a Python script is written to synchronize the two signals. This allows for the construction of the stress-strain curve for each tested specimen.

## 3. Results

This section presents the results obtained from the tensile tests on the specimens, using a testing speed of 2 mm/min. For each test conducted, the machine provides a data file, which is analyzed to generate the curves that are presented. Among the processed data provided by the machine are:

### Tensile Tests

**Figure 2:**
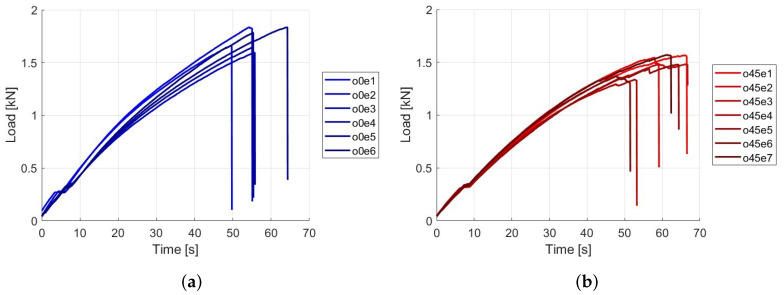
Load versus time results recorded by the load cell during tensile tests: (**a**) load response for fiber specimens oriented at 0 degrees, and (**b**) load response for fiber specimens oriented at 45 degrees.

The curves are generated using the data from the load and time, creating a force vs. time graph to compare the load at which the specimen’s fracture and the time taken. Six valid tests (i.e., fracture in the central area of the specimen and not in the grip section) were considered for the specimens oriented at 0 degrees, and seven valid tests were considered for the specimens oriented at 45 degrees. See the results in Figure 2. The nomenclature for each specimen type is o0ei, with i = 1…6 for Type A specimens, and o45ei, with i = 1…7 for Type B specimens.

The results presented in Table 2 show the maximum load and fracture time for two types of samples, labeled as Type A. The tests show that the maximum load the samples can endure ranges from 1.5920 kN to 1.8328 kN, with the highest load recorded in test 5 and the lowest in test 3. The fracture time varies between 49.67 s and 64.25 s, indicating some samples can withstand higher loads but fracture faster, while others last longer under similar loads. The average maximum load is 1.7201 kN with a standard deviation of 0.1 kN, suggesting moderate variability in the results.

The results presented in Table 3 highlight the maximum load and fracture time for Type B samples across multiple tests. The maximum load endured by the samples ranges from 1.3376 kN to 1.5704 kN, with the highest load recorded in test 7 and the lowest in test 4. The time to reach maximum load varies from 47.87 s to 66.29 s. Additionally, the samples experience a load drop, ranging from 1.2992 kN to 1.5632 kN, with the corresponding load drop time varying from 51.41 s to 66.65 s. The average maximum load is 1.4726 kN with a standard deviation of 0.09 kN, and the average load drop is 1.4558 kN with a standard deviation of 0.1 kN.

**Figure 3:**
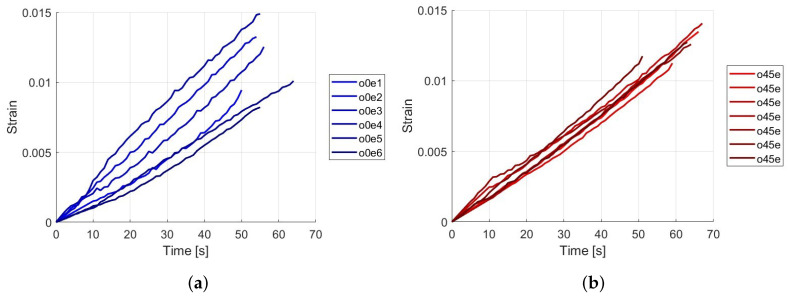
Strain versus time curves for the principal strain obtained through digital image correlation during tensile tests. (**a**) Fiber specimens oriented at 0 degrees. (**b**) Fiber specimens oriented at 45 degrees.

The curves are generated using the data from strain and time, creating a strain vs. time graph to compare the maximum strain at which the specimen fractures and the time taken. The tests were conducted on six specimens for each type to contrast the results. See these results in Figure 3.

The results presented in Table 4 show the maximum strain and fracture time for two types of samples, labeled as Type A. The tests show that the maximum strain the samples can endure ranges from 0.0049 to 0.0148, with the highest strain recorded in test 4 and the lowest in test 1. The fracture time varies between 50 s and 64 s, indicating that some samples can withstand higher strains but fracture faster, while others last longer under similar strains. The average maximum strain is 0.0106 with a standard deviation of 0.003, suggesting moderate variability in the results.

The results presented in Table 5 highlight the maximum strain and fracture time for Type B samples across multiple tests. The maximum strain endured by the samples ranges from 0.0100 to 0.0140, with the highest strain recorded in test 3 and the lowest in test 4. The time to reach maximum strain varies from 50 s to 67 s. The average maximum strain is 0.0122 with a standard deviation of 0.001.

**Figure 4:**
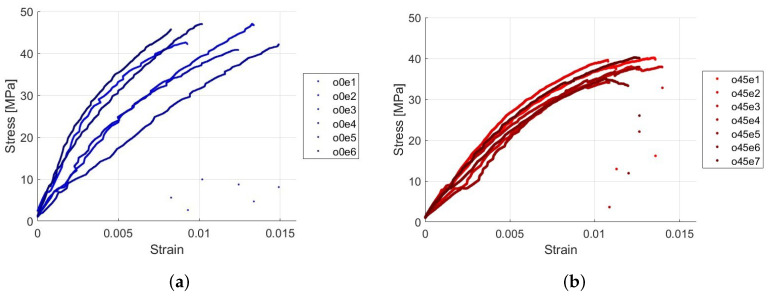
Stress versus strain curves recorded during tensile tests. (**a**) fiber specimens oriented at 0 degrees, (**b**) fiber specimens oriented at 45 degrees.

The curves are generated using the data from stress and strain, creating a stress vs. strain graph to compare the stress at which the specimen fractures and the corresponding strain. The tests were conducted on six specimens for each type to contrast the results. See these results in Figure 4.

The results presented in Table 6 show the stress and strain for the samples labeled as Type A. The tests indicate that the stress the samples can endure ranges from 40.82 MPa to 46.99 MPa, with the highest stress recorded in test 5 and the lowest in test 3. The strain varies between 0.0049 and 0.0148, indicating that some samples can withstand higher stresses but exhibit lower strains. In samples with this type of orientation, the strain behavior relative to the stress shows greater dispersion. The average stress is 44.10 MPa with a standard deviation of 2.67 MPa, and the average strain is 0.0106 with a standard deviation of 0.003.

The results presented in Table 7 highlight the stress and strain for Type B samples across multiple tests. The stress endured by the samples ranges from 35.15 MPa to 40.26 MPa, with the highest stress recorded in test 7 and the lowest in test 6. The strain varies from 0.0100 to 0.0140. The average stress is 38.46 MPa with a standard deviation of 1.91 MPa, and the average strain is 0.0122 with a standard deviation of 0.001.

## 4. Discussion

The results of the load versus time and strain versus time show the following: First, it is evident that the samples with fibers oriented at 0 degrees (Type A) exhibit a higher maximum load capacity, with an average of 1.7201 kN, compared to 1.4726 kN for the 45-degree oriented samples (Type B). This suggests that the 0-degree orientation allows for greater tensile strength, likely because the fibers are aligned with the direction of the applied load, optimizing the load transfer through the fibers. The comparison of maximum strain and fracture time between the 0-degree and 45-degree fiber orientations, as presented in Table 3 and Table 4, reveals important differences in the mechanical behavior of the composite materials. For the 0-degree orientation (Table 3), the maximum strain varies from 0.0049 to 0.0148, with an average strain of 0.0106. The corresponding fracture times range from 50 to 64 s. Notably, the data indicates that samples with higher strains, such as those in tests 2, 3, and 4, tend to fracture within a relatively narrow time frame (between 54 and 56 s). This suggests that, despite the variations in strain, the time to fracture remains somewhat consistent, likely due to the direct alignment of the fibers with the loading direction, which limits the material’s ability to deform before failure. In contrast, the 45-degree orientation (Table 4) exhibits a slightly higher average maximum strain of 0.0122, with values ranging from 0.0100 to 0.0140. The fracture times for these samples are generally longer, ranging from 50 to 67 s. The higher strains and extended fracture times in the 45-degree samples suggest that this orientation allows for greater deformation before failure, likely due to the distribution and less direct alignment of the fibers relative to the applied load. This distribution of forces across the fibers leads to a more gradual failure process, as evidenced by the more consistent strain values and longer fracture times. Overall, the 45-degree orientation appears to provide a better deformation response, allowing the material to endure higher strains and sustain loads for longer periods before fracturing. This behavior contrasts with the 0-degree orientation, where the material exhibits higher stiffness, leading to quicker failure once the strain threshold is reached.

The results of the tests show significant variations in force, deformation, and stress between the two types of samples (A and B), providing a detailed insight into their mechanical behavior under loading conditions.

In the Type A samples, the maximum force ranges from 1.5920 kN to 1.8328 kN, while in the Type B samples it varies from 1.3376 kN to 1.5704 kN. This difference indicates that Type A samples have a slightly higher load-bearing capacity compared to Type B samples. Additionally, the fracture time for Type A samples varies between 49.67 and 64.25 s, whereas for Type B samples it ranges from 47.87 to 66.65 s. The variation in fracture times suggests a difference in failure resistance between the two types, with Type B samples exhibiting slightly more consistent temporal behavior.

For deformation and time, the maximum strain in Type A samples varies from 0.0049 to 0.0148, with corresponding fracture times ranging from 50 to 64 s. In contrast, Type B samples show strain values ranging from 0.0100 to 0.0140, with fracture times between 50 and 67 s. These differences indicate that Type A samples experience a wider range of deformations under load before fracturing, while Type B samples demonstrate greater consistency in the maximum strain reached before failure.

The stress and strain results reveal that Type A samples endure stresses ranging from 40.82 MPa to 46.99 MPa, with corresponding strains between 0.0049 and 0.0148. Conversely, Type B samples endure stresses ranging from 35.15 MPa to 40.26 MPa, with strains between 0.0100 and 0.0140. This indicates that Type A samples can withstand higher stress levels before reaching their maximum strain compared to Type B samples.

**Figure 5:**
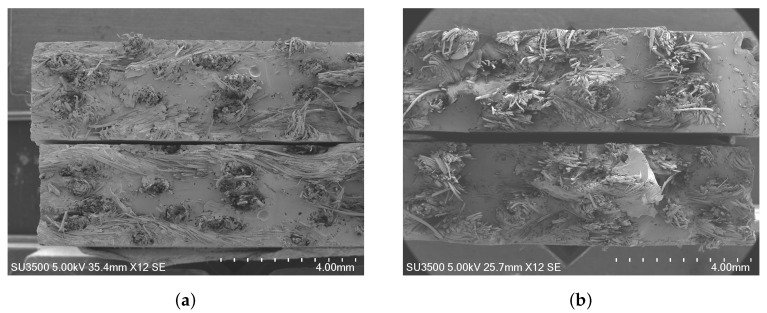
Fracture surfaces observed using SEM after tensile tests. (**a**) Fiber specimens oriented at 0 degrees. (**b**) Fiber specimens oriented at 45 degrees.

A key observation is the difference in load drop behavior between the two sample types. In Type A samples, the load drop is immediate upon reaching the maximum load, attributed to both the fiber and the polymer matrix cutting simultaneously. In contrast, Type B samples show that the fibers do not cut immediately, causing the load not to drop abruptly for a short period of time. This difference in behavior is clearly illustrated in Figure 5, where the fracture mechanisms of both sample types are visible. Figure 5a, corresponding to the sample with fiber orientation at 0 degrees, shows a fiber almost cut flush with the polymer matrix. Furthermore, it can be seen that the transverse fiber acts like an imperfection since the fracture occurred precisely in that place. On the other hand, Figure 5b, corresponding to the sample with fiber orientation at 45 degrees, clearly shows the dispersion of fibers.

Upon reviewing the tables, we found significant differences between the two fiber orientations in terms of their average values. The average maximum load for the 0-degree samples is 1.7201 kN, which is 16.8% higher than the 1.4726 kN for the 45-degree samples. Similarly, the 0-degree samples show an average maximum stress of 44.10 MPa, 14.7% higher than the 38.46 MPa observed in the 45-degree samples. Conversely, the 45-degree samples exhibit a 15.1% higher average maximum strain (0.0122) compared to the 0-degree samples (0.0106). Additionally, the fracture times are consistently longer in the 45-degree samples, with an average increase of 6.0% to 7.8%. These differences highlight the fiber orientation effect on the mechanical properties of the material, underscoring the need for a detailed statistical analysis to fully characterize this variability.

**Figure 6:**
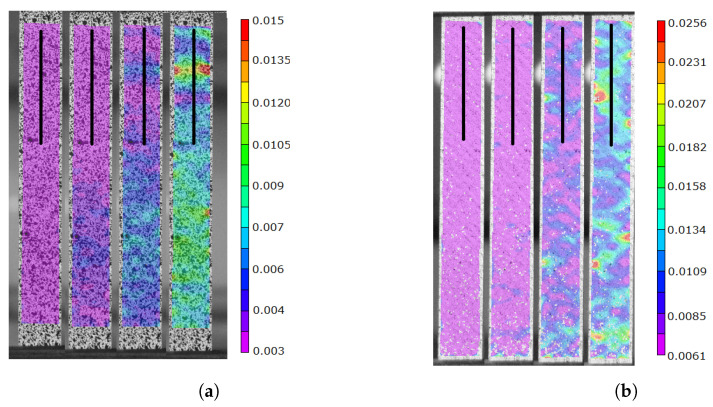
Axial strain field measured using DIC at various time steps during the tensile test (**a**) 0 degrees at 15, 25, 35 and 50 s, (**b**) 45 degrees at 15, 35, 50 and 61 s.

**Figure 7:**
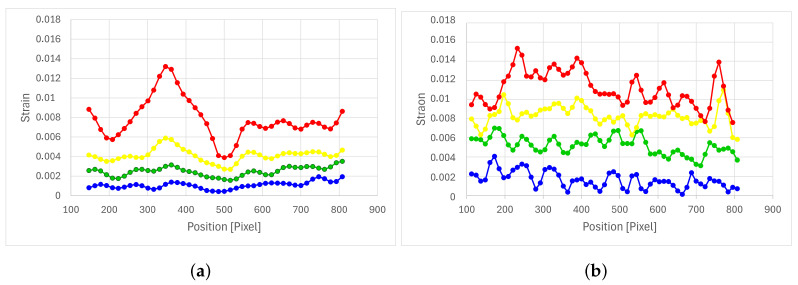
Axial strain profile (**a**) 0 degrees at 15 (blue), 25 (green), 35 (yellow) and 50 (red) seconds, and (**b**) 45 degrees at 15 (blue), 35 (green), 50 (yellow) and 61 (red) seconds.

Figure 6 shows images of the deformation process of the samples. Figure 6a corresponds to samples with fiber orientation at 0 degrees, and Figure 6b corresponds to samples with fiber orientation at 45 degrees. In both images, it can be seen that the deformation field is noticeably different. The sample at 0 degrees shows a deformation field with zones of concentration aligned with the fiber orientation. Figure 7a shows the deformation profile corresponding to the deformations extracted from the black line, where the distribution of deformations in the axial direction can be observed. In the case of the sample at 45 degrees, a deformation field with a better distribution of deformations can be observed, also aligned with the fiber orientation. Figure 7b shows the deformation profile corresponding to the deformations extracted from the black line, where the distribution of deformations in the axial direction can be observed. Figure 7a,b clearly show that the distribution of the fibers is responsible for the distribution of the deformations.

## 5. Conclusions

The experimental exploration of the mechanical response of a polymeric matrix reinforced with jute fiber with two different orientations was analyzed. Tensile tests, digital image correlation techniques and SEM analyses were carried out. The following conclusions were obtained:

- Tensile tests revealed that fibers oriented at 0 degrees contribute to the strengthening of the polymeric matrix, resulting in reduced strain under applied load. However, fibers oriented transversely do not contribute to matrix strengthening and instead act as imperfections. This effect is also observed in samples with diagonal fiber orientation, where greater deformation occurs under applied load, but with less contribution to the strengthening of the polymeric matrix.
- The diagonal fiber orientation facilitates a more uniform distribution of deformations, effectively preventing their localization and reducing the risk of polymer matrix fracture. In contrast, samples with fibers oriented at 0 degrees exhibit a higher concentration of deformations, making the matrix more susceptible to fracture.

## Figures and Tables

**Table 1:** Amounts of each component used in grams to fabricate the specimens.

Orientation	Jute Fiber	Resin	Accelerator	Catalyst
$0^{\circ }$	36 g	165 g	0.66 g	4.95 g
$45^{\circ }$	36 g	165 g	0.66 g	4.95 g

**Table 2:** Fracture time and load at 0 degrees.

Type	Test	Max. Load (kN)	Fracture Time (s)
A	1	1.6472	49.67
	2	1.8224	50.01
	3	1.5920	55.73
	4	1.6440	55.19
	5	1.8328	64.25
	6	1.7824	55.31
	$\bar{X}$	1.7201 (0.1)	

**Table 3:** Fracture time and load at 45 degrees.

Type	Test	Max. Load (kN)	Max. Load Time (s)	Load Drop (kN)	Load Drop Time (s)
B	1	1.5656	66.29	1.5512	66.47
	2	1.5448	57.95	1.4904	58.79
	3	1.4824	59.27	1.4776	66.65
	4	1.3376	52.73	1.3296	53.15
	5	1.4360	56.51	1.4792	64.31
	6	1.3712	47.87	1.2992	51.41
	7	1.5704	60.95	1.5632	62.27
	$\bar{X}$	1.4726 (0.09)		1.4558 (0.1)	

**Table 4:** Fracture time and maximum strain at 0 degrees.

Type	Test	Max. Strain	Fracture Time (s)
A	1	0.0049	50
	2	0.0132	54
	3	0.0125	56
	4	0.0148	55
	5	0.0101	64
	6	0.0082	55
	$\bar{X}$	0.0106 (0.003)	

**Table 5:** Fracture time and maximum strain at 45 degrees.

Type	Test	Max. Strain	Fracture Time (s)
B	1	0.0134	66
	2	0.0112	59
	3	0.0140	67
	4	0.0100	50
	5	0.0125	64
	6	0.0117	51
	7	0.0127	63
	$\bar{X}$	0.0122 (0.001)	

**Table 6:** Stress and strain values at 0 degrees.

Type	Test	Max. Tensile Stress [MPa]	Fracture Strain
A	1	42.23	0.0049
	2	46.72	0.0132
	3	40.82	0.0125
	4	42.15	0.0148
	5	46.99	0.0101
	6	45.70	0.0082
	$\bar{X}$	44.10 (2.67)	0.0106 (0.003)

**Table 7:** Stress and strain values at 45 degrees.

Type	Test	Max. Tensile Stress [MPa]	Fracture Strain
B	1	40.14	0.0134
	2	39.61	0.0112
	3	38.01	0.0140
	4	39.29	0.0100
	5	36.82	0.0125
	6	35.15	0.0117
	7	40.26	0.0127
	$\bar{X}$	38.46 (1.91)	0.0122 (0.001)

## Data Availability

The data supporting the findings of this study are available from the corresponding author under request.
